# Impaired Attentional Control in Pedophiles in a Sexual Distractor Task

**DOI:** 10.3389/fpsyt.2016.00193

**Published:** 2016-12-02

**Authors:** Kirsten Jordan, Peter Fromberger, Jakob von Herder, Henrike Steinkrauss, Rebekka Nemetschek, Joachim Witzel, Jürgen L. Müller

**Affiliations:** ^1^Department for Forensic Psychiatry and Psychotherapy, Clinic of Psychiatry and Psychotherapy, University Medical Center, Georg-August-University of Göttingen, Göttingen, Germany; ^2^Central State Forensic Psychiatric Hospital of Saxony-Anhalt, Uchtspringe, Germany

**Keywords:** pedophilia, attentional control, executive functions, eye movements, distractors

## Abstract

Pedophilic disorder, a subtype of paraphilia, is defined as a recurrent sexual interest in prepubescent children, which is characterized by persistent thoughts, fantasies, urges, sexual arousal, or behavior. Besides a deviant sexual preference, sexual preoccupation was found to be a dynamic risk factor for reoffending. Thus, it is conceivable that sex offenders and especially sex offenders against children have difficulties to control their responses to sexual stimuli. In the current study pedophiles, forensic and non-forensic control subjects had to solve a cognitive task, while sexual distractors were presented simultaneously. This kind of task also requires control functions. Therefore, data were analyzed with respect to attentional control while comparing eye movements toward sexual distractors and toward the cognitive task. We were mainly interested in how early (fixation latency) and late (relative fixation time) attentional processes were allocated to both, the cognitive target stimuli and the sexual distractors. Pedophiles demonstrated significantly lower attentional control in the sexual distractor task than both control groups (non-pedophiles). They showed a shorter fixation latency and longer fixation time for sexual distractors than non-pedophiles. Furthermore, pedophiles demonstrated a longer fixation latency and shorter fixation time for cognitive target stimuli. For classification analyses, an attentional control index (ACI) was built, i.e., the difference between eye movements on cognitive target stimuli and sexual distractors. For the ACI of early attentional processes, i.e., fixation latency, a good classification between pedophiles and non-pedophiles was found. We assumed that the measured attentional control represents inhibitory executive functions, specifically interference control. Further studies should examine if low attentional control in pedophiles is due to low motivation to solve the task or rather to a lack of ability to control attention with respect to sexual and/or neutral distractors. Prospectively, this design could be useful to generate hypotheses about clinical important aspects of controllability, the capacity of self-control, and the severity of a paraphilic disorder.

## Introduction

Pedophilic disorder, a subtype of paraphilia, is defined as a recurrent sexual interest in prepubescent children, which is characterized by persistent thoughts, fantasies, urges, sexual arousal, or behavior (1). A deviant sexual preference is one of the major predictors for sexual recidivism of sexual offenders (2). It is conceivable that sex offenders and especially sex offenders against children have difficulties to control their responses to sexual stimuli. Sexual preoccupation was found to be a dynamic risk factor for reoffending in sexual offenders (2). Seto and Fernandez (3) applied the 16-item Stable-2000 to identify different dynamic risks groups among 419 adult male sexual offenders. Among others, they found a sexually deviant group who scored high on deviant sexual interests, sex drive/preoccupation, emotional identification with children, and child molester attitudes.

Sex offender treatment programs rely on cognitive or behavioral interventions to reduce the risk of recidivism. Cognitive behavioral therapies are intended to change internal processes – thoughts, beliefs, emotions, physiological arousal – alongside changing overt behavior, such as social skills or coping behavior (4). Control functions, as a part of executive functions, are of importance for those behaviors. Offenders learn to monitor and control thoughts, feelings, and behaviors associated with offending, in order to adopt alternative ways of coping with deviant sexual thoughts and desires.

Hence, it is of interest to explore the behavior of sex offenders in response to sexual stimuli, especially if they have to concentrate on a different task. Working on a cognitive task while being potentially distracted by sexual stimuli, needs control functions in order to focus on the cognitive task. Therefore, in the current study we were interested in attentional control functions in sex offenders while applying a sexual distractor task. Some aspects are of importance regarding the processing of sexual stimuli and control functions in sex offenders.

### Sexuality, Attention, and Control Functions

From an evolutionary perspective, biological significant stimuli are processed with increased priority to maximize the efficiency of reacting. Thus, early attentional processes allow for a high level of processing even before these stimuli are perceived consciously (5). For threat stimuli, like spiders or snakes, these survival facilitating mechanisms, i.e., a prioritized and rapid processing, are well known [e.g., Ref. (6, 7)]. Similar mechanisms have been proposed for sexual stimuli, which are linked to opportunities for reproduction (8). The information processing approach of Spiering and Everaerd (9) assumes that sexually relevant features of a stimulus are preattentively selected and automatically induce focal attention to these sexually relevant aspects. The focal attention on the sexually relevant stimulus induces conscious appraisal of these stimulus aspects. If the stimuli are in accordance with the sexual scripts of the explicit memory, the viewer classifies the stimulus as sexually relevant. This induces a conscious experience of sexual arousal.

Considering the automatic as well as controlled processing of sexual stimuli, it can be assumed that the processing of sexual features which are presented along with a cognitive task should interfere with the processing of this task. This effect is also known as the sexual content-induced-delay [SCID (10)]. Based on the underlying, broader concept of limited attention capacity during controlled information processing (11), sexual features and the cognitive stimulus compete for the limited attention capacity. This leads to an inference between the processing of the cognitive task and the processing of the sexual features. Due to the evolutionary importance of sexual features, it has been proposed that performance in the cognitive task should be impaired. Support for this assumption comes from various studies. Within the forensic field, studies applying the choice reaction time task [CRT (12)] or the pictorial-modified Stroop task [P-MST (13)] showed, though not consistently, a prolonged reaction time (RT) when a sexually relevant stimulus was presented along with a cognitive task, compared to a sexually non-relevant stimulus or a neutral stimulus.

Most approaches which applied the concept of the SCID, used measurement of performance (RT, errors) to assess the processing delay. However, this parameter could only deliver information about the endpoint of the distraction process. Continuously measured eye movements could enhance the information about the cognitive processes, especially if it is possible to distinguish between eye movements toward (cognitive) target stimuli and (sexual) distractors. Using eye movements, also early and late attentional processes could be measured, as proposed by Spiering and Everaerd (9). In an initial orientation approach, Fromberger et al. (14) used the number of first fixations toward sexual stimuli to measure early attentional processes and relative fixation time for sexual stimuli to measure late attentional processes. For heterosexual subjects they found that relative fixation time was significantly longer and the number of first fixation was higher for sexually preferred stimuli than for sexually non-preferred stimuli (14).

Interestingly, several studies used eye movements to examine executive functions. The anti-saccade task, for instance, has been used to assess inhibitory cognitive control functions. In this task, subjects have to suppress an automatic response to look at a peripherally presented target, in order to initiate a motor command to look away from the target (15). Patients with dysfunctions in frontal lobes or basal ganglia exhibit difficulties to perform this task correctly, e.g., patients with schizophrenia, neurodegenerative dementia, or Parkinson’s disease (16, 17). The measurement of eye movements in a computerized visual search task, which is based on the Trail Making Test (18) seems also to be suitable to characterize changes in executive functioning with and without transcranial magnetic stimulation (19).

Thus, sexual stimuli are processed at automatic as well as at controlled level and eye movements seem to be suitable to assess control functions regarding the processing of those stimuli.

### Executive Functions in Sexual Offenders

Executive functions are important for daily life. Using executive functions, we can stay focused, resist temptations, or think before acting. Core executive functions comprise inhibitory control, working memory, and cognitive flexibility (20). Regarding sex offenders, inhibitory functions are of special interest, since they include response inhibition and interference control. Response inhibition can be defined as inhibition on a behavioral level, i.e., self-control and discipline to resist temptations or to resist acting impulsively (20). Interference control includes the inhibition on the attentional and cognitive level, i.e., selective and focused attention and the inhibition of thoughts and memories. Stone and Thompson (21) examined 63 male sexual offenders, including offenders against adults and children and exhibitionists, applying a large neuropsychological test battery. Even though some sex offenders scored within normal range, an overall test of means demonstrated significant differences from normative scores. Suchy et al. (22) reported executive weaknesses in pedophilic and non-pedophilic child molesters compared to non-offender controls. In a further study, the overall executive profiles of child molesters were different from that of non-sexual offenders (23). They, for instance, performed more poorly on inhibition (interference control in the Color–Word Interference Test). Pedophilic and non-pedophilic child molesters also seem to be impaired in simple response inhibition (24). They showed more errors, but no longer RTs in a go/no-go task compared to healthy controls and non-sexual offenders. By comparing pedophilic subjects and healthy controls in a go/no-go task, Habermeyer et al. (25) found significantly longer response times for no-go trials and slightly more commission errors in pedophiles. Based on a recent meta-analysis on neuropsychological functions, Joyal et al. (26) concluded that sex offenders against children tended to have impaired higher order executive functions than sex offenders against adults (especially in terms of cognitive flexibility and deduction). Otherwise, they were significantly better than sex offenders against adults regarding the control of internal interference and verbal fluency. Sex offenders against adults tended to score similarly to non-sex offenders, but with lower scores in verbal fluency and inhibition (26). Thus, although the picture has to be completed, we sum up that executive functions, especially inhibitory control functions, seem to be impaired in sexual offenders if compared to general population.

### Aim of the Study

Working on a cognitive task while being potentially distracted by sexual stimuli, needs control functions in order to focus on the cognitive task. Those functions seem to be impaired in sex offenders and especially in sex offenders against children. From a diagnostic and therapeutic perspective, the measurement of control functions with respect to sexual stimuli seems to be important. Therefore, in the current study, we were interested in attentional control functions in a sexual distractor task. Data were analyzed with respect to attentional control while comparing eye movements toward sexual distractors and toward cognitive task. We were mainly interested in how early (fixation latency) and late (relative fixation time) attentional processes were allocated to cognitive target stimuli and sexual distractors, respectively. Data were further analyzed to prove if eye movement variables would coincide with subjects’ group statuses on a better-than-chance level. We asked whether these attentional control processes would differ between groups with respect to distractor category.

## Materials and Methods

### Participants

Altogether, data of 22 male pedophilic subjects, 7 male forensic inpatients without any history of sexual assault against children, and 50 male healthy subjects were analyzed. The current study was part of a larger project (27–29). In a previous analysis, data of the current experiment were analyzed with respect to sexual interest (29). Data, just of the non-forensic control group, are presented in von Herder et al. (30).

Healthy subjects were recruited *via* a notice posted on bulletin boards in Göttingen and on inquiry at a police-officer school. Pedophilic subjects and forensic control subjects were recruited at high-security, forensic-psychiatric hospitals. Inclusion criteria for the pedophilic group were a cross-validated diagnosis of pedophilia (ICD-10 F65.4) by two experienced clinicians and mandatory hospitalization under treatment order for a child-sexual-abuse offense (validated through forensic records). Inclusion criteria for the forensic control group were the absence of a diagnosis of pedophilia, no child-sexual-abuse offense, and mandatory hospitalization under treatment order for an adult-sexual-abuse offense (validated through forensic records). Inclusion criteria for the healthy, non-forensic control group were the absence of any psychiatric illnesses, deviant sexual fantasies, or behavior (validated by an extensive psychiatric and sexual anamnesis conducted in a systematic oral interview about the case history of the subject by one experienced clinician). Exclusion criteria (especially for the inpatient groups) were an acute psychotic episode or substance abuse during the previous month, no agreement between the two clinicians with respect to the diagnosis of pedophilia, or incapability or refusal to sign informed consent. Due to these specifications and other conditions (e.g., no informed consent, technical problems) 36 (3 pedophiles and 33 non-pedophiles) out of 65 screened forensic inpatients had to be excluded for the current analysis. Two healthy subjects (out of 52) had to be excluded from the analysis due to technical problems.

Table 1 summarizes the characteristics of the three groups of participants with regard to sexual orientation and ICD-10 diagnosis. For the pedophilic group and the forensic control group, sexual orientation was assessed based on the victims’ gender. Sexual orientation of non-forensic controls was assessed with the Kinsey scale (31), accepting only ratings from 0 to 1 (exclusively and predominantly heterosexual) or 5 to 6 (predominantly or exclusively homosexual). As shown in Table 1, the three groups were not homogeneous with regard to their sexual orientation and their psychiatric diagnosis.

**Table 1:** **Detailed characteristics of the subject groups**.

Number of subjects	Pedophiles (*N* = 22)	Forensic controls (*N* = 7)	Non-forensic controls (*N* = 50)	Test statistic^a^
**Sexual orientation**				
Heterosexual	9 (40.9%)	7 (100%)	34 (68%)	**χ^2^(2) = 9.28, *p* = 0.010**
Homosexual	7 (31.8%)	0 (0%)	16 (32%)	χ^2^(2) = 3.16, *p* = 0.210
Bisexual	6 (27.3%)	0 (0%)	0 (0%)	**χ^2^(2) = 16.82, *p* < 0.001**
**ICD-10 diagnosis^b^**				
Pedophilia (F65.4)	22 (100%)	0 (0%)	0 (0%)	**χ^2^(2) = 79.00, *p* < 0.001**
Substance abuse/dependence (F10–F19)	9 (40.9%)	2 (28.6%)	0 (0%)	**χ^2^(2) = 22.71, *p* < 0.001**
Schizophrenia (F20–F29)	3 (13.6%)	2 (28.6%)	0 (0%)	**χ^2^(2) = 11.20, *p* = 0.004**
Neurotic disorders (F40–F49)	2 (9.1%)	0 (0%)	0 (0%)	χ^2^(2) = 5.32, *p* = 0.07
Personality disorders (F60–F69)	9 (40.9%)	4 (57.1%)	0 (0%)	**χ^2^(2) = 20.06, *p* < 0.001**
Mental disorders (F70–F79)	3 (13.6%)	0 (0%)	0 (0%)	**χ^2^(2) = 8.10, *p* = 0.018**
Developmental disorders (F80–F89)	1 (4.5%)	0 (0%)	0 (0%)	χ^2^(2) = 2.62, *p* = 0.27
Behavioral disorders with onset in childhood (F90–F99)	1 (4.5%)	0 (0%)	0 (0%)	χ^2^(2) = 2.62, *p* = 0.27
**Demographic data^c^**				
Age, years (SD)	42.09 (10.92)	34.86 (14.28)	25.38 (7.39)	***F*(2, 76) = 26.05, *p* < 0.001, η^2^ = 0.41**
Intelligence, overall mean IQ (SD)	76.52 (16.65)	78.14 (7.14)	117.88 (11.03)	***F*(2, 75) = 95.60, *p* < 0.001, η^2^ = 0.72**
Hospitalization, month (SD)	121.82 (68.02)	116.00 (112.62)	0 (0)	***F*(2, 76) = 58.41, *p* < 0.001, η^2^ = 0.61**
**Basic mental rotation performance^d^**				
Reaction time (ms), mean (SD)	4530.89 (1376.01)	5553.38 (999.65)	3885.75 (1021.22)	**Group: *F*(2, 71) = 4.81, *p* = 0.011, η^2^ = 0.12**
				**Age: *F*(1, 71) = 9.95, *p* = 0.002, η^2^ = 0.12**
				IQ: *F*(1, 71) = 0.02, *p* = 0.890, η^2^ = 0.00
				Hosp: *F*(1, 71) = 0.76, *p* = 0.386, η^2^ = 0.01
Error rate (%), mean (SD)	37.65 (18.75)	32.15 (9.06)	13.38 (9.24)	Group: *F*(2, 71) = 0.25, *p* = 0.779, η^2^ = 0.01
				Age: *F*(1, 71) = 3.23, *p* = 0.077, η^2^ *=* 0.04
				**IQ: *F*(1, 71) = 16.01, *p* < 0.000, η^2^*=* 0.18**
				Hosp. *F*(1, 71) = 0.63, *p* = 0.432, η^2^ *=* 0.01

*Shown are sexual orientations, ICD-10 diagnosis, demographic data, and basic mental rotation performance*.

*Percentage within groups is given*.

*Bold fonts are used for significant results*.

*^a^Test statistic for sexual orientation and ICD-10 diagnosis: chi-square test. Test statistic for demographic data: univariate general linear model (GLM) with the factor group. Test statistic for the basic mental rotation performance: univariate general linear model with the factor group and covariates age, intelligence, and hospitalization*.

*^b^Only those ICD-diagnosis are presented, which were appropriate for at least one subject. Participants with an F10–F19 ICD-10 diagnosis had no active substance abuse at least in the last month. Participants with an F20–F29 ICD-10 diagnosis had no acute psychotic episode at least during the last month. Personality disorders (without F65.4) were assessed with SKID-II (32)*.

*^c^Age: Post hoc pairwise comparisons: non-forensic control group vs. pedophiles, p < 0.001, non-forensic control group vs. forensic control group, p = 0.04. Intelligence: Post hoc pairwise comparisons: non-forensic control group vs. pedophiles, p < 0.00, non-forensic control group vs. forensic control group, p < 0.001. Hospitalization reflects the overall time duration of the subjects in forensic hospitals. Post hoc pairwise comparisons: non-forensic control group vs. pedophiles, p < 0.001, non-forensic control group vs. forensic control group, p < 0.001*.

*^d^Basic mental rotation performance: reaction time, main effect group: post hoc pairwise comparisons: pedophiles vs. forensic control group, p = 0.012*.

The pedophilic participants demonstrated a median score of 5.00 (range: 2–5) on the Screening Scale for Pedophilic Interests, which identified the group as a high-risk sample with respect to recidivism (33, 34). The pedophilic group had been convicted for sexually abusing an average of 6.05 children (SE = 1.03, range: 1–22). Child victims were, on average, 9.03 years of age (SE = 0.47 years, range: 3.50–12.50 years).

**Figure 1:**
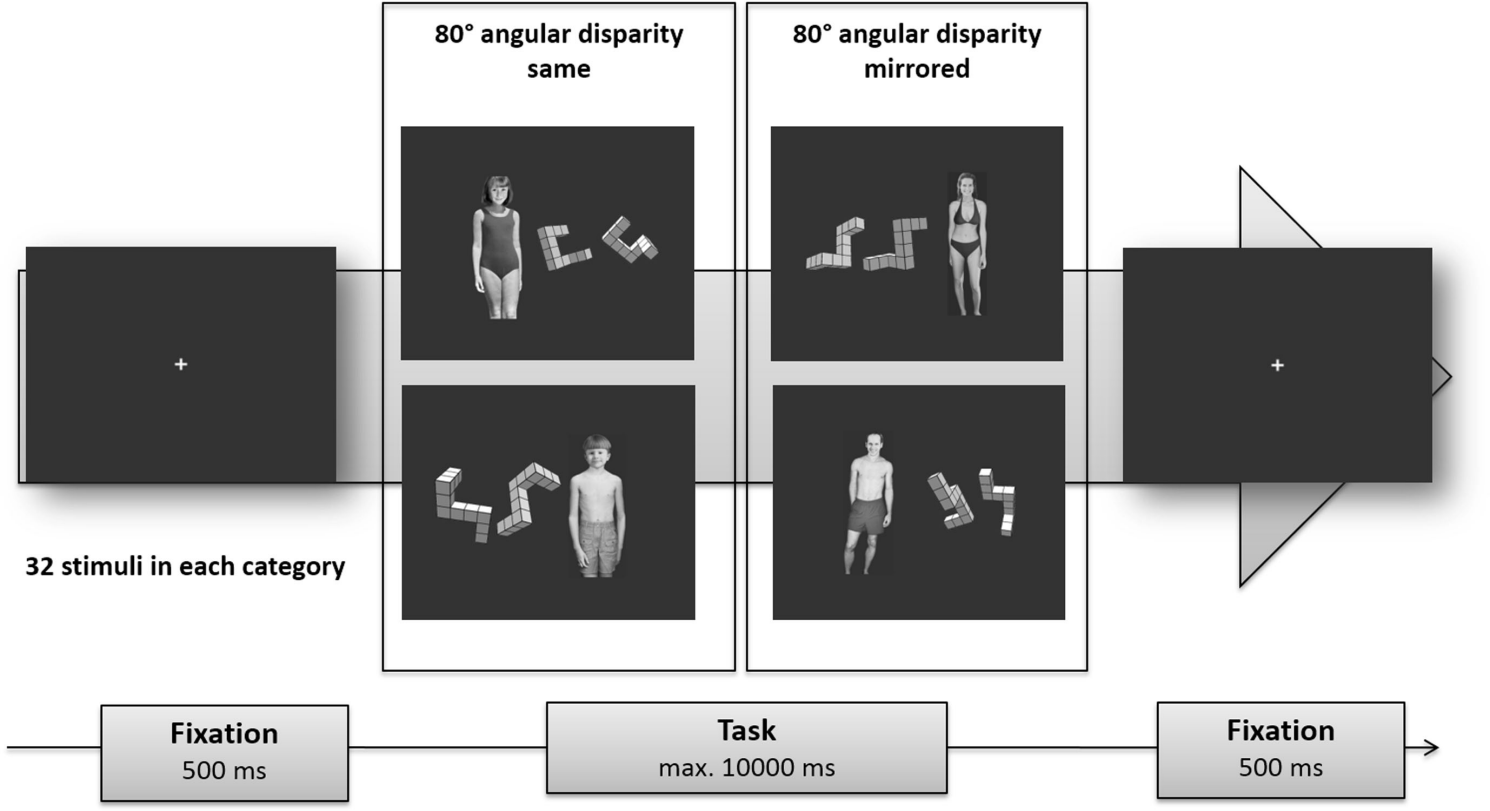
**Experimental design [see also Ref**. (29)**]**. Given are examples for each condition. For each mental rotation task, one sexual distractor simultaneously was presented, a girl, boy, woman, or man (note that these stimuli were not included in the main task). Sexual distractor stimuli were taken from the NRP-set (36). Mental rotation stimuli were selected from the set developed by Paschke et al. (37). For details, see Section “Materials and Methods.”

Intelligence was assessed by the Wechsler Adult Intelligence Scale (German version) (35). The basic mental rotation performance was assessed using the same task which was used in the sexual distractor task, but without sexual distractors (see below and Figure 1). Groups differed with respect to age, intelligence, hospitalization, and basic mental rotation performance (see Table 1). Mostly, the healthy control group differed from both forensic groups. With respect to the potential significant influence of age, intelligence, and hospitalization on task performance, these factors were included as covariates in all statistical analyses comparing groups.

All participants had normal or corrected-to-normal visual acuity. All of them provided written informed consent in accordance with the Declaration of Helsinki, before participating in the experiment. The study was approved by the Ethics Committee of the Medical Faculty of Georg-August-University of Göttingen.

### Sexual Distractor Task

#### Mental Rotation Stimuli

The classical mental rotation of three-dimensional figures was first described by Shepard and Metzler (38). Meantime, mental rotation of three-dimensional figures has been applied in a broad range of psychological and neuroscience research (39, 40). Typically, pairs of two- or three-dimensional cube figures are presented, either identical and rotated or mirrored and rotated. The angle of rotation between the figures ranges from 20° to 180°. Subjects have to decide if the two figures are identical or not. Considering the large empirical knowledge about cognitive processes and the possibility to systematically modulate the difficulty of the mental rotation task (41, 42), this task seems to be suitable for an application in a new design to measure sexual preference and attentional control processes under cognitive demand.

In the current study, pairs of three-dimensional cube figures were presented, identical and rotated (*n* = 32), or mirrored and rotated (*n* = 32). In order to increase the number of stimuli, each pair of mental rotation figures were presented twice, once on the right side of the screen and once on the left side, resulting in a total of 128 stimuli. Stimuli were taken from a larger stimulus set which was developed by Paschke et al. (37). In our study, we chose a fixed angular disparity of 80° that had been associated with moderate error rates of about 10% in the study by Paschke et al. (37).

#### Sexual Distractor Stimuli

Sexual stimuli were taken from the Not-Real-People (NRP) picture set (36). The NRP picture set consists of a total of 160 colored images of partially dressed and nude people of both genders at five different stages of pubertal development, according to Tanner’s categorization (43, 44). The images are non-pornographic in terms of explicit sexual poses or sexual activity. In this study, 64 nude male and female images were used. Male and female stimuli of Tanner stages 1 and 2 were combined to make up the distractor categories “boy” and “girl.” “Woman” and “man” distractor categories were comprised of Tanner stages 4 and 5. Tanner 3 images were not used in this study. Using each distractor stimulus twice, once as a mirrored copy and once in its original orientation, we achieved a total of 64 stimuli, 16 per distractor category.

#### Combination of Mental Rotation Stimuli and Sexual Distractors

Stimulus displays consisted of a horizontal presentation of the mental rotation stimulus and a distractor stimulus out of one of the four categories woman, man, girl, or boy (see Figure 1). Rotation stimuli and distractors were positioned in a way that their center points lay 12° of visual angle apart, assuming a viewing distance of 70 cm. The sides of display as well as the combinations of distractor categories and rotation stimuli were balanced across trials. Every distractor was seen twice in combination with different types of rotation tasks resulting in 128 trials altogether.

To control low-level visual features such as color, luminance, contrast, and visual complexity (45), all images were converted into gray scale and processed with a self-developed Matlab script (Matlab Version 7.6.0, MathWorks Inc.) to even out significant differences in luminance and contrast. The backgrounds of images were replaced by a monochrome gray. As the mean file size of files in JPEG format is correlated positively with their visual complexity (46), a comparison between mean byte number between distractor categories helped to further rule out confounding bottom-up attentional bias (14). Stimuli were presented on a 19-inch TFT flat screen (1280 × 1024 pixels, 75 Hz).

### Eye Tracking Device

Eye movements were measured using an SMI iView X RED eye tracker (SensoMotoric Instruments GmbH, Berlin, Germany) in combination with an iViewX workstation (spatial solution < 0.1° of visual angle, temporal solution 60 Hz, gaze position accuracy of < 0.4° of visual angle). Eye movements were reordered with a video-based infrared eye camera using corneal reflection and dark pupil method. The SMI RED system is a contact free, remote controlled eye tracking device with automatic eye and head tracker assuring that slight head movements are automatically compensated (within a range of approximately 40 cm). Although, it was not necessary to immobilize the head of the participants, we needed to ensure that they did not move out of the compensable range. Therefore, we asked the subjects to rest their chins on their non-dominant hands.

### Procedure

Participants were seated in a quiet room facing the monitor at eye level at a viewing distance of 27.6″ from the monitor. To introduce subjects to the sexual distractor task, a training experiment was conducted. During training only clothed sexual distractor stimuli and pairs of mental rotation figures were presented, which were not included in the main task. A feedback was given regarding the speed and accuracy of the answer after each test trial. The experiment itself was divided into 4 blocks of 32 trials which were each preceded by a 9-point calibration of the eye tracker and after which participants could rest for as long as they felt the need to. Before each trial, a fixation cross appeared in the center of the screen to ensure a central gaze position at the beginning of a trial. Stimulus presentation was triggered if participants continuously fixated the cross for 500 ms as indicated by a red circle appearing around it. Subjects responded *via* the press of one of two buttons to judge the stimulus parity. Each trial ended either after button press or after 10 s. During the whole experiment RT, button presses and eye movements were recorded.

### Data Analysis

#### Behavioral Data

In the sexual distractor task, mean RT for correct answers and error rate were calculated with respect to sexual distractor categories which were simultaneously presented with the mental rotation stimulus. RT values below 150 ms were excluded from analysis.

#### Eye Movements

Raw eye movements were analyzed using BeGaze 3 (Sensomotoric Instruments GmbH, Berlin, Germany) to identify fixations [see also Ref. (14)]. Fixations were defined as periods of relative stability of gaze position within a field of 1° of visual angle for at least 100 ms (47). Different areas of interest (AOIs) were marked to analyze visual attention to the different stimuli. Each pair of mental rotation figures equated to one AOI, and each image of a woman, girl, boy, or man served as one AOI. Two eye movement parameters were analyzed. The fixation latency was defined as the duration from stimulus onset to the first fixation within a specific AOI. Fixation latency is thought to represent attentional bias owed to early, automatic shifts in attention, especially if it represents the first fixation in a trial (27, 48). In contrast, fixation time is known to reflect controlled, sustained attention, e.g., late, mostly top-down endogenous control of attention [e.g., Ref. (48)]. Fixation time was measured as relative fixation time, i.e., the sum of fixation duration of all fixations located within the relevant AOI, divided by the whole presentation time of each task. The latter was restricted either by the response time of the participant or by the maximum presentation time of the task, 10 s.

#### Statistical Analyses

All statistics were performed with IBM SPSS Statistics 22.0 [IBM Corp. and other(s) 1989, 2013, NY, USA]. To control for the unequal distribution of general sexual orientations within the three groups, only the sexually relevant images with respect to gender were analyzed (12, 27). Hence, for heterosexual participants, only images of females (girls and women) and for homosexual participants, only images for males (boys and men) were included. For bisexual participants, images of both males and females were included in the analysis. This strategy resulted in two sexual age categories: child and adult. To emphasize that these sexual stimuli served as distractors in the sexual distractor task, they were referred to as “sexual distractors.” Besides this “sexual distractor category,” an additional variable “stimulus type” was introduced. This was done in order to compare eye movements toward the sexual distractors, i.e., the sexual stimuli and toward mental rotation stimuli. Thus, two stimulus types were used in the analysis: sexual distractors and mental rotation stimuli.

Behavioral data (RT, error rate) were analyzed applying 3 × 2[group (non-forensic control, forensic control, pedophile) × distractor category (child, adult)] mixed design general linear models (GLMs) with the covariates age, intelligence, and hospitalization.

To analyze eye movement differences within groups, 2 × 2[distractor category (child, adult)] × 2[stimulus type (mental rotation stimulus, sexual distractor)] repeated measure GLM were applied. Group differences regarding eye movements were examined applying a 3 × 2 × 2[group (non-forensic control, forensic control, pedophile) × distractor category (child, adult) × stimulus type (mental rotation stimulus, sexual distractor)] mixed design GLMs with the covariates age, intelligence, and hospitalization. Bonferroni-corrected two-tailed *post hoc* tests were applied. Significant interactions were further analyzed by univariate repeated measure GLMs.

#### Attentional Control Index

Receiver operating characteristic (ROC) analyzes were performed in order to determine how well the eye movements to the different stimulus types differentiate between groups. Classifier performance was measured by the area under the curve (AUC). In order to do so, an attentional control index (ACI) was computed for each subject, i.e., the difference between the eye movements toward mental rotation stimuli and toward sexual distractors. For each eye movement parameter, this computation resulted in two variables. The “ACI-fixation latency-adult” represented the difference between mean fixation latency to mental rotation figures with a simultaneously presented adult sexual distractor and mean fixation latency to the adult sexual distractor itself. The “ACI-fixation latency-child” meant the difference between mean fixation latency to mental rotation figures with a child sexual distractor and mean fixation latency to the child sexual distractor itself. Similar computations were done for relative fixation time. A low ACI for the fixation latency represented low mean fixation latency toward mental rotation stimuli compared to higher mean fixation latency toward sexual distractors. Thus, a low ACI represents a good attentional control regarding the fixation latency in this task. A high ACI for the relative fixation time stood for a long relative fixation time for mental rotation stimuli and a short relative fixation time for sexual distractors, thereby a good attentional control in this task. Univariate GLMs were computed to explore whether the ACI for the eye movements differentiated between the pedophiles and the non-pedophiles (i.e., both control groups). Only, if significant group differences were found, ROC-analyzes were added. The cutoff criterion was determined following the approach by Youden (49). Following this approach, the optimal cutoff point is the threshold that maximizes the distance to the identity (diagonal) line. The optimal criterion is defined as *y* = max (sensitivities + specificities).

## Results

### Mental Rotation Performance

Mixed design GLM for RT in the sexual distractor task yielded a significant main effect for the group but not for the distractor category (see Table 2). The covariates age of the subjects and hospitalization had a significant influence, but not intelligence. Pairwise comparisons demonstrated significantly lower RTs for the non-forensic control group compared to the forensic control group. No interactions were found. Concerning errors in the mental rotation task, the non-forensic control group exhibited lowest error rate (about 8%) compared to both forensic groups with error rates about 25–30%. These group differences could mainly be explained by differences regarding the covariates intelligence and age. The mixed design GLM revealed no main effect for the group. A significant influence of intelligence and age was demonstrated. No further significant main effects or interactions were found.

**Table 2:** **Mental rotation performance in the sexual distractor task with respect to the subject groups**.

Mental rotation task	Distractor category	Pedophiles (*N* = 22)	Forensic controls (*N* = 7)	Non-forensic controls (*N* = 50)	Test-statistic^a^ overall group differences	Test-statistic^b^ pedophiles vs. forensic controls	Test-statistic^b^ pedophiles vs. non-forensic controls	Test-statistic^b^ forensic controls vs. non-forensic controls
Reaction time (ms)	Adult distractor	4224.82 (1190.94)	4752.02 (1456.36)	2959.45 (1137.06)	**Group: *F*(2, 72) = 4.9, *p* = 0.010, η^2^ = 0.12**	*p* = 0.228	*p* = 0.144	***p* = 0.008**
Mean (SD)	Child distractor	4295.12 (1284.22)	4806.46 (1122.94)	2782.74 (1082.96)	Distractor category: *F*(1, 72) = 0.49, *p* = 0.484, η^2^ = 0.01			
					Group × distractor category: *F*(2, 72) = 0.038, *p* = 0.963, η^2^ = 0.001			
					**Age: *F*(1, 72) = 9.2, *p* = 0.003, η^2^ = 0.11**			
					Intelligence: *F*(1, 72) = 0.177, *p* = 0.675, η^2^ = 0.002			
					**Hospitalization: *F*(1, 72) = 5.05, *p* = 0.028, η^2^ = 0.07**			
Error rate (%)	Adult distractor	32.10 (18.83)	25.00 (10.97)	8.75 (7.36)	Group: *F*(2, 71) = 0.90, *p* = 0.409, η^2^ = 0.03	n.a.	n.a.	n.a.
Mean (SD)	Child distractor	29.47 (21.54)	22.32 (10.74)	6.19 (8.64)	Distractor category: *F*(1, 72) = 0.0, *p* = 0.99, η^2^ = 0.03			
					Group × distractor category: *F*(2, 72) = 1.08, *p* = 0.346, η^2^ = 0.03			
					**Age: *F*(1, 71) = 4.59, *p* = 0.036, η^2^ = 0.06**			
					**Intelligence: *F*(1, 72) = 20.62, *p* < 0.001, η^2^ = 0.22**			
					Hospitalization: *F*(1, 72) = 1.22, *p* = 0.274, η^2^ = 0.02			

*Results of the mixed design GLM are given*.

*Bold fonts are used for significant results*.

*^a^General linear models were applied with covariates age, intelligence, and hospitalization*.

*^b^*Post hoc* pairwise comparisons (Bonferroni-corrected)*.

### Eye Movements – Fixation Latency

**Figure 2:**
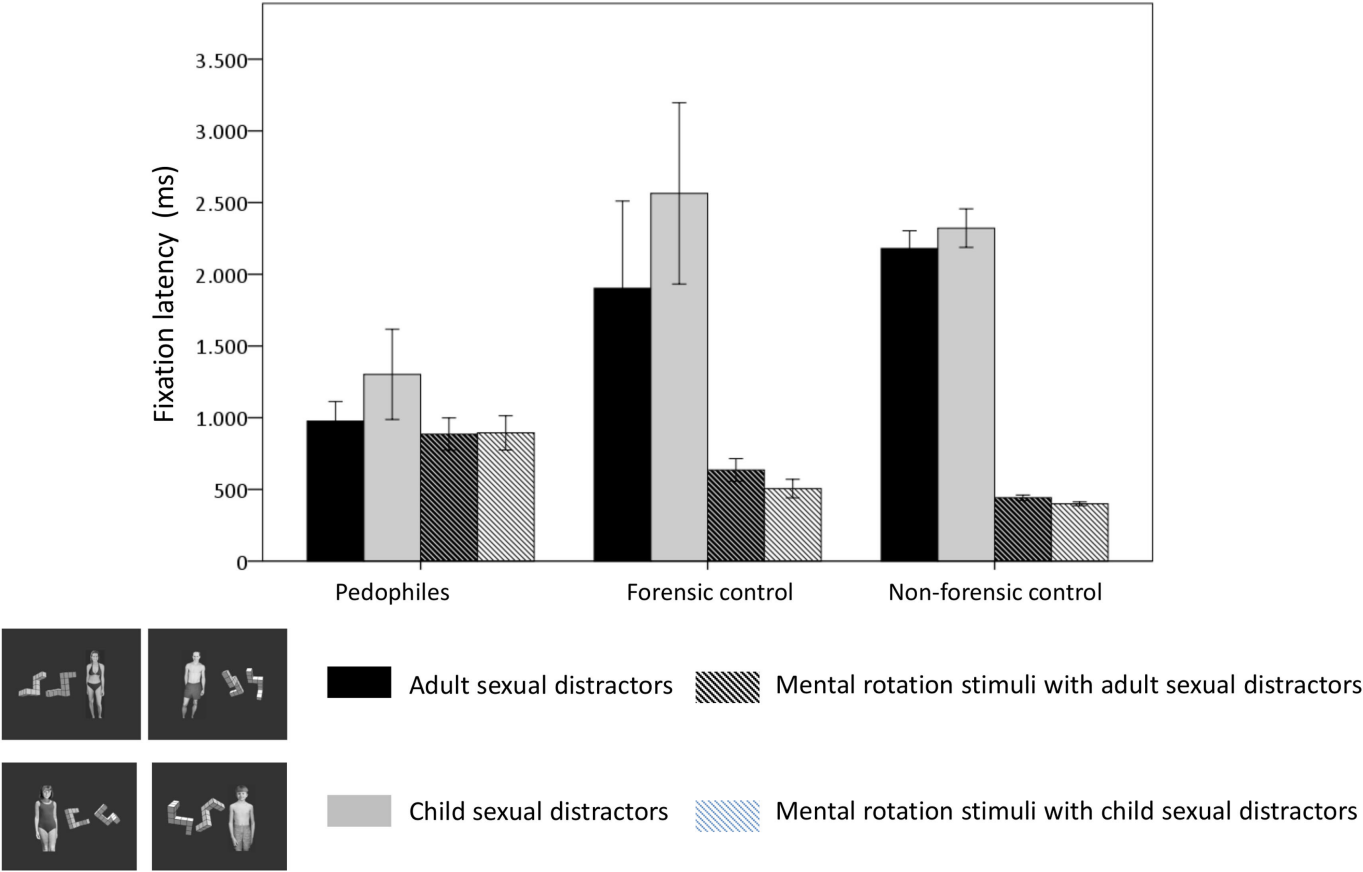
**Eye movements in the sexual distractor task: fixation latency**. Means and SEs are shown of the fixation latency for the first fixation with respect to stimulus type (single-colored: sexual distractors, striped: mental rotation figures) and distractor category (black/black-striped: adult, gray/gray-striped: child). This resulted in four different stimuli. Black bars: fixation latency for the adult sexual distractor. Gray bars: fixation latency for the child sexual distractor. Striped black bars: fixation latency for the mental rotation stimulus if the adult sexual distractor was simultaneously presented. Striped gray bars: fixation latency for the mental rotation stimulus if the child sexual distractor was simultaneously presented.

Figure 2 presents the means and SEs for the fixation latency in the three groups with respect to stimuli types (i.e., mental rotation stimulus, sexual distractor) and distractor categories (adult, child). Detailed statistical results of the repeated measure GLMs within groups are shown in Table S1 in Supplementary Material. Table 3 presents the results of the mixed design GLM for the comparison between groups with respect to fixation latency. The analysis for fixation latencies yielded a significant interaction between stimulus type and group. No further main effects or interactions were seen. None of the covariates had a significant influence. To disentangle the interaction effect, *post hoc* 3 × 2[group (non-forensic control, forensic control, pedophile) × distractor category (child, adult)] mixed design GLMs were applied for each stimulus type. The GLM for the stimulus type “sexual distractor” revealed a significant main effect for the group with shorter fixation latencies on sexual distractors in pedophiles compared to the forensic control group (see also Figure 2). No further main effects or interactions were found. None of the covariates had a significant influence. Similar results were found for the stimulus type “mental rotation stimulus.” The main effect group reached statistical significance. Pedophiles exhibited significant longer fixation latencies for mental rotation figures compared to both control groups.

**Table 3:** **Eye movements in the sexual distractor task with respect to subject group, distractor category, and stimulus type**.^a^

Eye movement parameter	3(group) × 2(distractor category) × 2(stimulus type) mixed design GLM	Stimulus type	*Post hoc* 3(group) × 2(distractor category) mixed design GLM	Test-statistic pedophiles vs. forensic controls^b^	Test-statistic pedophiles vs. non-forensic controls^b^	Test-statistic forensic controls vs. non-forensic controls^b^
Fixation latency	Group: *F*(2, 66) = 1.78, *p* = 0.174, η^2^ = 0.05	Sexual distractor	**Group: *F*(2, 66) = 3.59, *p* = 0.033, η^2^ = 0.10**	***p* = 0.042**	*p* = 0.517	*p* = 1.00
	Stimulus type: *F*(1, 66) = 0.68, *p* = 0.414, η^2^ = 0.01		Distractor category: *F*(1, 66) = 0.15, *p* = 0.704, η^2^ = 0.002			
	Distractor category: *F*(1, 66) = 0.22, *p* = 0.641, η^2^ = 0.003		Group × distractor category: *F*(2, 66) = 0.26, *p* = 0.773, η^2^ = 0.01			
	**Group × stimulus type: *F*(2, 66) = 5.73, *p* = 0.005, η^2^ = 0.15** Group × distractor category: *F*(2, 66) = 0.12, *p* = 0.887. η^2^ = 0.004		Covariates Age: *F*(1, 66) = 0.003, *p* = 0.955, η^2^ = 0.00 Intelligence: *F*(1, 66) = 0.40, *p* = 0.529, η^2^ = 0.01 Hospitalization: *F*(1, 66) = 0.51, *p* = 0.478, η^2^ = 0.01			
	Stimulus type × distractor category: *F*(1, 66) = 0.08, *p* = 0.775, η^2^ = 0.001 Stimulus type × distractor category × group: *F*(2, 66) = 0.46, *p* = 0.635, η^2^ = 0.01	Mental rotation stimulus	**Group: *F*(2, 72) = 10.81, *p* < 0.001, η^2^ = 0.23** Distractor category: *F*(1, 72) = 0.44, *p* = 0.509, η^2^ = 0.01	***p* = 0.009**	***p* < 0.001**	*p* = 0.417
	Covariates Age: *F*(1, 66) = 0.02, *p* = 0.882, η^2^ = 0.00 Intelligence: *F*(1, 66) = 0.50, *p* = 0.483, η^2^ = 0.01 Hospitalization: *F*(1, 66) = 1.7, *p* = 0.193, η^2^ = 0.03					
			Group × distractor category: *F*(2, 72) = 1.42, *p* = 0.248, η^2^ = 0.04			
			Covariates Age: *F*(1, 72) = 0.10, *p* = 0.749, η^2^ = 0.001 Intelligence: *F*(1, 72) = 0.00, *p* = 0.985, η^2^ = 0.00 Hospitalization: *F*(1, 72) = 2.12, *p* = 0.143, η^2^ = 0.03			
Relative fixation time	Group: *F*(2, 72) = 0.72, *p* = 0.492, η^2^ = 0.02	Sexual distractor	**Group: *F*(2, 72) = 4.6, *p* = 0.013, η^2^ = 0.11**	***p* = 0.014**	*p* = 0.364	*p* = 1.00
	**Stimulus type: *F*(1, 72) = 7.91, *p* = 0.006, η^2^ = 0.10**		Distractor category: *F*(1, 72) = 0.04, *p* = 0.847, η^2^ = 0.001			
	**Pairwise comparison: *p* < 0.001**		Group × distractor category: *F*(2, 72) = 0.92, *p* = 0.403, η^2^ = 0.03			
	Distractor category: *F*(1, 72) = 0.63, *p* = 0.429, η^2^ = 0.01		Covariates Age: *F*(1, 72) = 0.15, *p* = 0.687, η^2^ = 0.002 Intelligence: *F*(1, 72) = 0.00, *p* = 0.973, η^2^ = 0.000 Hospitalization: *F*(1, 72) = 0.15, *p* = 0.702, η^2^ = 0.002			
	**Group × stimulus type: *F*(1, 72) = 5.45, *p* = 0.006, η^2^ = 0.13**					
	Group × distractor category: *F*(2, 72) = 2.42, *p* = 0.096, η^2^ = 0.063	Mental rotation stimulus	**Group: *F(*2, 72) = 3.87, *p* = 0.025, η^2^ = 0.10**	***p* = 0.037**	*p* = 0.280	*p* = 1.00
	Stimulus type × distractor category: *F*(1, 72) = 0.28, *p* = 0.597, η^2^ = 0.004		Distractor category: *F*(1, 72) = 0.59, *p* = 0.446, η^2^ = 0.01			
	Stimulus type × distractor category × group: *F*(2, 72) = 1.80, *p* = 0.173, η^2^ = 0.05		Group × distractor category: *F*(2, 72) = 2.72, *p* = 0.073, η^2^ = 0.07			
	Covariates Age: *F*(1, 72) = 2.68, *p* = 0.106, η^2^ = 0.04 Intelligence: *F*(1, 72) = 0.48, *p* = 0.489, η^2^ = 0.007 Hospitalization: *F*(1, 72) = 0.08, *p* = 0.779, η^2^ = 0.001		Covariates Age: *F*(1, 72) = 1.48, *p* = 0.227, η^2^ = 0.02 Intelligence: *F*(1, 72) = 0.41, *p* = 0.526, η^2^ = 0.01 Hospitalization: *F*(1, 72) = 0.001, *p* = 0.978, η^2^ = 0.00			

*Results of the statistical analyses are given*.

*Bold fonts are used for significant results*.

*^a^First, a 3(group) × 2(distractor category) × 2(stimulus type) mixed design GLM was applied. In order to disentangle the interaction group × stimulus type a *post hoc* 3(group) × 2(distractor category) mixed design GLM was performed*.

*^b^Pairwise Bonferroni-corrected comparisons*.

### Eye Movements – Relative Fixation Time

**Figure 3:**
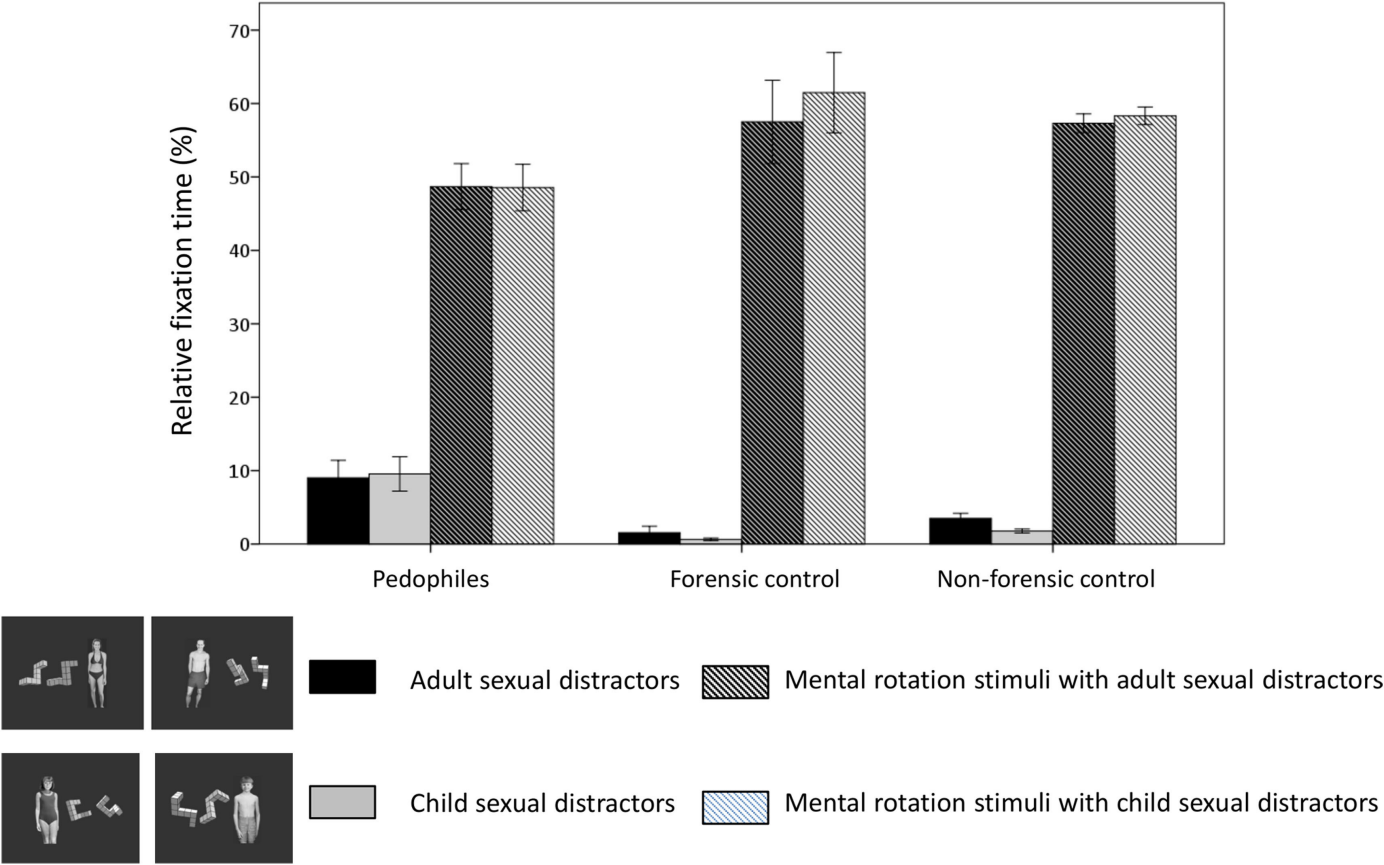
**Eye movements in the sexual distractor task: relative fixation time**. Means and SEs are shown of the relative fixation time with respect to stimulus type (single-colored: sexual distractors, striped: mental rotation figures) and distractor category (black/black-striped: adult, gray/gray-striped: child). This resulted in four different stimuli. Black bars: fixation latency for the adult sexual distractor. Gray bars: fixation latency for the child sexual distractor. Striped black bars: fixation latency for the mental rotation stimulus if the adult sexual distractor was simultaneously presented. Striped gray bars: fixation latency for the mental rotation stimulus if the child sexual distractor was simultaneously presented.

Figure 3 presents the means and SEs for relative fixation time in the three groups with respect to the two stimuli types (i.e., mental rotation stimulus, sexual distractor) and two distractor categories (adult, child). Detailed statistical results for the analysis among groups are presented in Table S2 in Supplementary Material. Table 3 presents the detailed statistical results of the mixed design GLM for the comparisons between groups with respect to relative fixation time. The analysis yielded a significant main effect for stimulus type with longer fixation times for mental rotation figures compared to sexual distractors (see also Figure 3). Furthermore, there was a significant interaction between stimulus type and group. The interaction Distractor category × Group was significant only by trend. No further main effects or interactions were seen. None of the covariates had a significant influence. To unbound the interaction between stimulus type and group, *post hoc* 3 × 2[group (non-forensic control, forensic control, pedophile) × distractor category (child, adult)] mixed design GLMs were applied for each stimulus type (for detailed statistical results, see Table 3). The analysis for stimulus type “sexual distractor” resulted in a significant group effect with a longer relative fixation time to sexual distractors in pedophiles compared to the forensic control group. We found no further main effects or interactions. None of the covariates had a significant influence. The GLM for the stimulus type “mental rotation stimulus” yielded a significant main effect for the group. Pedophiles viewed the mental rotation figures significantly shorter than did subjects of the forensic control group. A small interaction in terms of a trend was seen between the distractor category and group.

### Attentional Control Index and Discrimination Accuracy

In order to determine how well the eye movements toward stimulus types could differentiate between groups we computed an ACI (see Data Analysis). Furthermore, we combined the groups to compare the pedophiles (*n* = 21) with non-pedophiles (both control groups, *n* = 57) regarding the attentional control.

#### ACI for Fixation Latency

**Figure 4:**
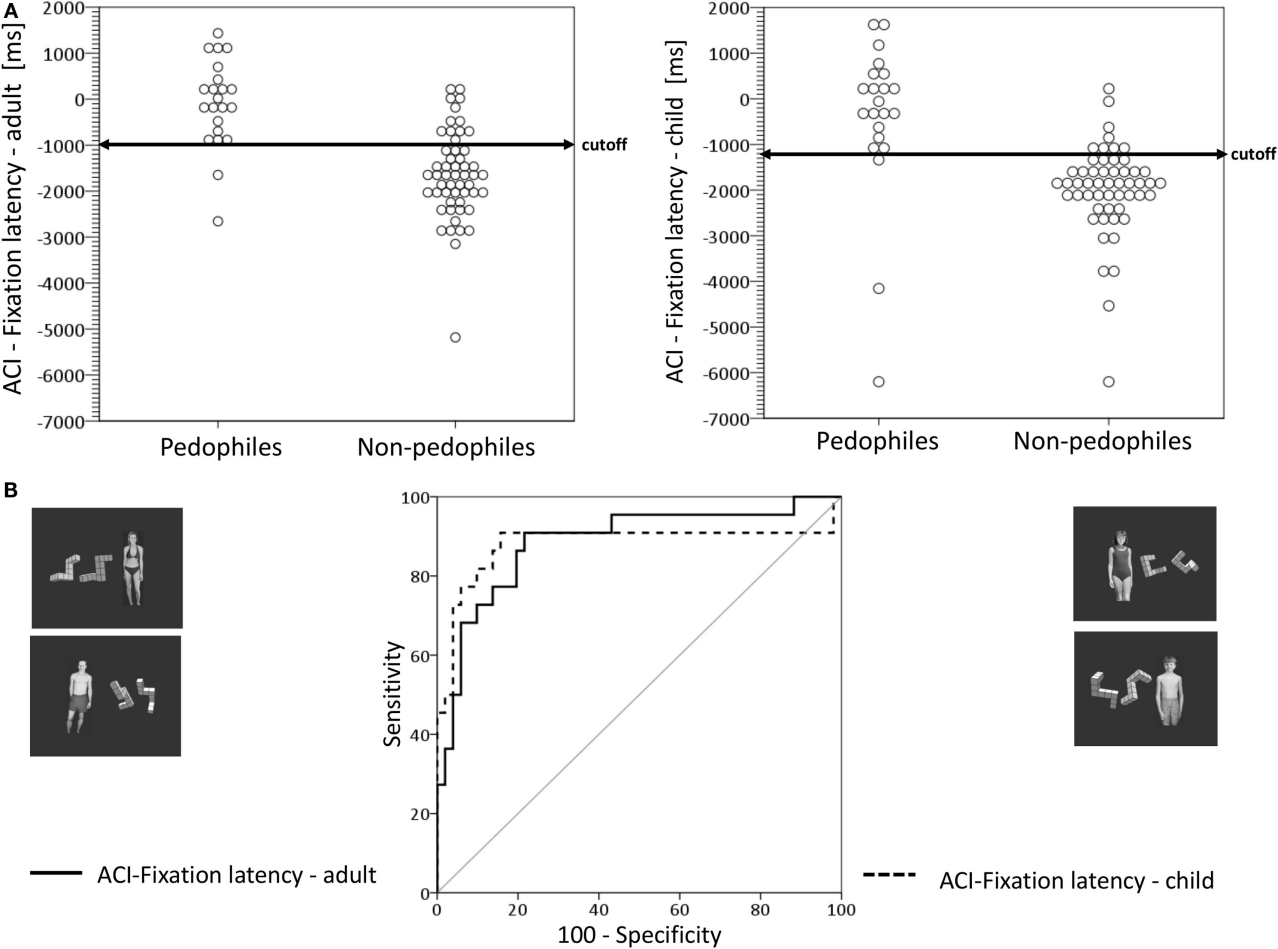
**Classifier performance of the attentional control index (ACI) for the fixation latency with respect to distractor category**. **(A)** Left: dot diagrams represent the individual attentional control index (ACI) value for each subject by subject group for the conditions if an adult sexual distractor was presented. Arrow represents the cutoff value of −999.37 ms. **(A)** Right: dot diagrams represent the individual attentional control index (ACI) value for each subject by subject group for the conditions if an child sexual distractor was presented. Arrow represents the cutoff value of −1264.33. **(B)** Receiver operating curve (ROC) of the ACI for the fixation latency plots the sensitivity vs. false-positive rate (1-specificity) as a variation of the cutoff value.

The univariate GLM for the ACI with respect to adult distractors and the appropriate mental rotation figures (ACI-fixation latency-adult) revealed a significant main effect for the group with a higher ACI for pedophiles compared to non-pedophiles (see Figure 4 and Table 4). None of the covariates had a significant influence. This result could indicate a significantly weaker control of eye movements in pedophiles compared to non-pedophiles. The ACI-fixation latency-adult discriminated between pedophiles and non-pedophiles with high accuracy. The ACI differed between pedophiles and non-pedophiles with a sensitivity of 90.9% (probability that a pedophile will be correctly classified as pedophilic). The specificity was 77.4%, i.e., the probability that a non-pedophile will be correctly classified a non-pedophilic.

**Table 4:** **Overview over the ACI for fixation latency and relative fixation time with respect to pedophilic and non-pedophilic subjects**.

	ACI-fixation latency – adult^a^ (ms)	ACI-fixation latency – child^a^ (ms)	ACI-relative fixation time – adult (%)	ACI-relative fixation time – child (%)
**ACI**				
Pedophiles, mean (SD), *n* = 22	−90.20 (967.36)	−407.90 (1747.56)	39.68 (22.87)	39.01 (23.30)
Non-pedophiles, mean (SD), *N* = 57	−1655.59 (967.77)	−1992.47 (1009.59)	54.10 (12.48)	57.07 (9.92)
Test statistic^b^	**Group: *F*(1, 69) = 10.10, *p* = 0.001, η^2^ = 0.13**	**Group: *F*(1, 69) = 13.91, *p* < 0.001, η^2^ = 0.17**	**Group: *F*(1, 73) = 6.99, *p* = 0.010, η^2^ = 0.09**	**Group: *F*(1, 73) = 14.27, *p* < 0.001, η^2^ = 0.16**
	***Post hoc*: *p* = 0.002**	***Post hoc*: *p* < 0.001**	***Post hoc*: *p* = 0.01**	***Post hoc*: *p* < 0.001**
	Age: *F*(1, 69) = 0.24, *p* = 0.624, η^2^ = 0.004	Age: *F*(1, 69) = 0.37, *p* = 0.54, η^2^ = 0.005	Age: *F*(1, 73) = 0.52, *p* = 0.475, η^2^ = 0.007	Age: *F*(1, 73) = 0.47, *p* = 0.493, η^2^ = 0.006
	Intelligence: *F*(1, 69) = 0.45, *p* = 0.507, η^2^ = 0.006	Intelligence: *F*(1, 69) = 0.04, *p* = 0.847, η^2^ = 0.001	Intelligence: *F*(1, 73) = 0.07, *p* = 0.792, η^2^ = 0.001	Intelligence: *F*(1, 73) = 0.01, *p* = 0.917, η^2^ = 0.000
	Hospitalization: *F*(1, 69) = 0.05, *p* = 0.833, η^2^ = 0.001	Hospitalization: *F*(1, 69) = 0.05, *p* = 0.828, η^2^ = 0.001	Hospitalization: *F*(1, 73) = 0.003, *p* = 0.959, η^2^ = 0.000	Hospitalization: *F*(1, 73) = 0.06, *p* = 0.815, η^2^ = 0.001
**ROC**				
AUC, *p*	**AUC = 0.877, *p* < 0.001**	**AUC = 0.883, *p* < 0.001**	**AUC = 0.702, *p* = 0.006**	**AUC = 0.739, *p* = 0.001**
95% CI	0.783–0.970	0.765–1.000	0.565–0.839	0.597–0.891
Cutoff	−999.37 ms	−1264.33 ms	49.88%	49.48%
Sensitivity (%)	90.9	90.9	71.9	84.2
Specificity (%)	77.4	84.9	63.6	63.6

*Results of GLM and receiver operating characteristics (ROC)-curves are given. Computation of ACI is based on the difference between mental rotation figures and the adult resp. child distractors (please see also notes below)*.

*Bold fonts are used for significant results*.

*ACI-fixation latency – adult: difference between fixation latency to mental rotation figures with an adult sexual distractor and fixation latency to the adult sexual distractor itself*.

*ACI-fixation latency – child: difference between fixation latency to mental rotation figures with a child sexual distractor and fixation latency to the child sexual distractor itself*.

*ACI-relative fixation time – adult: difference between relative fixation time for the mental rotation figures with an adult sexual distractor and relative fixation time for the adult sexual distractor itself*.

*ACI-relative fixation time – child: difference between relative fixation time for the mental rotation figures with a child sexual distractor and relative fixation time for the child sexual distractor itself*.

*^a^Number of subjects varied according to available data*.

*^b^Univariate GLM with age, intelligence, and hospitalization as covariates. Bonferroni-corrected post hoc test were applied*.

Regarding the ACI for child sexual distractors and the appropriate mental rotation figures (ACI-fixation latency-child) the GLM yielded significant group differences with a significant higher ACI in pedophiles compared non-pedophiles. None of the covariates had a significant influence. The ACI discriminated with high accuracy between pedophiles and non-pedophiles (sensitivity: 90.9%, specificity: 84.9%).

#### ACI for Relative Fixation Time

**Figure 5:**
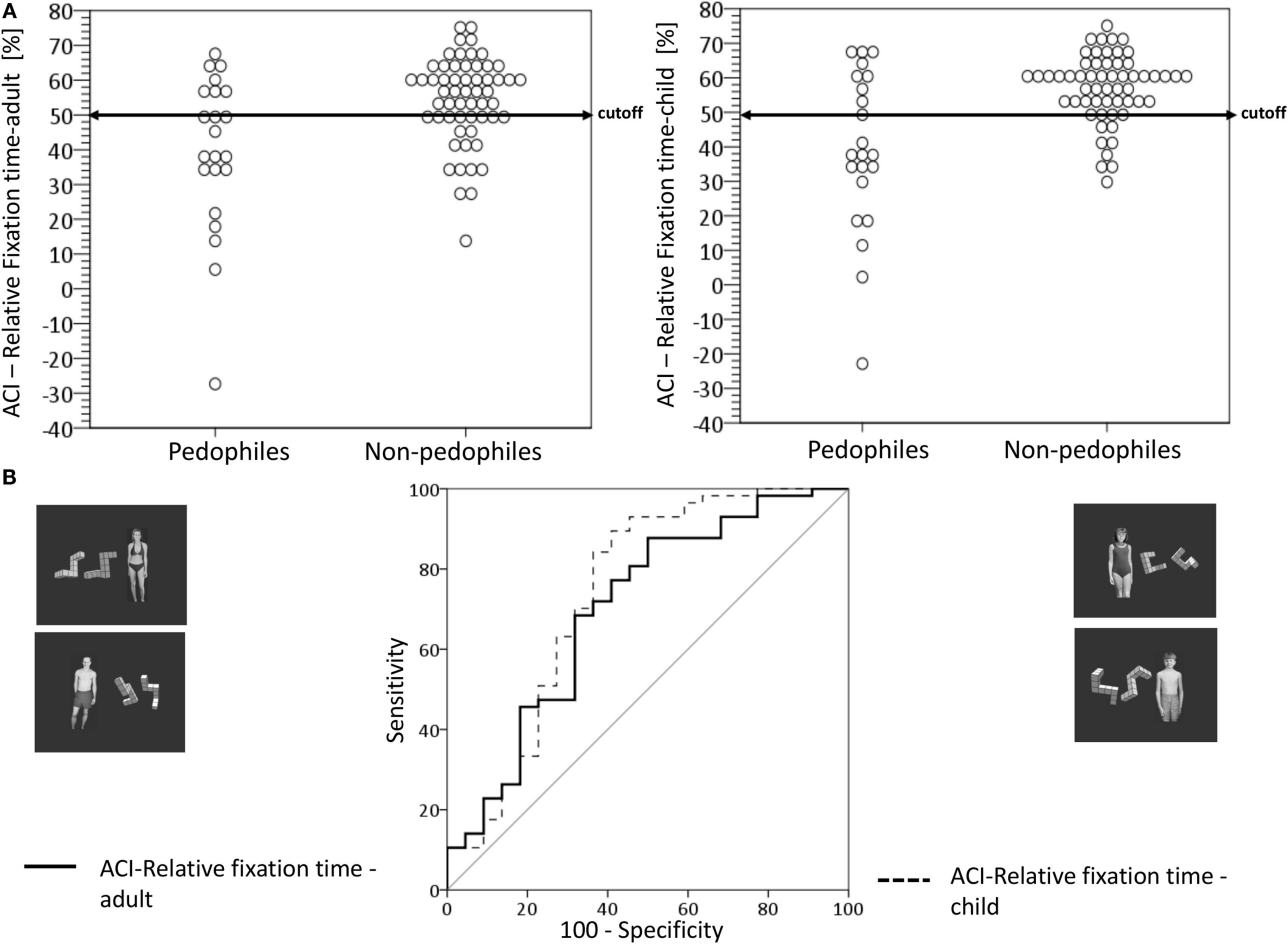
**Classifier performance of the attentional control index (ACI) for the relative fixation time with respect to distractor category**. **(A)** Left: dot diagrams represent the individual attentional control index (ACI) value for each subject by subject group for the conditions if an adult sexual distractor was presented. Arrow represents the cutoff value of 49.88%. **(A)** Right: dot diagrams represent the individual attentional control index (ACI) value for each subject by subject group for the conditions if an child sexual distractor was presented. Arrow represents the cutoff value of 49.48%. **(B)** Receiver operating curve (ROC) of the ACI for the relative fixation time plots the sensitivity vs. false-positive rate (1-specificity) as a variation of the cutoff value.

The application of a univariate GLM for the ACI of relative fixation time for adult distractor and the appropriate mental rotation figures (ACI-fixation time-adult) resulted in a significant main effect for the group with a lower ACI for pedophiles compared to non-pedophiles (see Figure 5 and Table 4). None of the covariates had a significant influence. This could indicate a lower attentional control in pedophiles than in non-pedophiles in the sexual distractor task. Results of the ROC-analysis showed that the ACI-fixation time-adult discriminated between pedophiles and non-pedophiles with a moderate accuracy. The ACI differentiated between pedophiles and non-pedophiles with a sensitivity of 71.9% (probability that a pedophile will be correctly classified as pedophilic). The specificity, i.e., the probability that a non-pedophile will be correctly classified as non-pedophilic, was 63.6%. Similar results were received for the ACI-fixation time-child with a significant group effect. Pedophiles demonstrated a lower ACI than non-pedophiles. None of the covariates had a significant influence. The ROC-analysis yielded a moderate discrimination accuracy between pedophiles and non-pedophiles (specificity: 84.2%, specificity: 63.6%).

## Discussion

The aim of the current study was to analyze attentional control processes in a sexual distractor task, in pedophiles, forensic control patients, and healthy subjects. We were especially interested in the interaction between the processing of cognitive target stimuli and sexual distractors. Results revealed that in our task pedophiles exhibited significantly lower attentional control than both control groups. This result could be seen in analyses of early as well as late attentional control processes.

### Attentional Control under Cognitive Load – Early Attentional Processes

Analyses for the ACI-fixation latency revealed a significantly higher ACI (for both distractors) for pedophiles compared to non-pedophiles. This high ACI in pedophiles could indicate a significantly inferior control of early attentional processes in pedophiles. Control groups exhibited large differences between short fixation latencies toward mental rotation figures and long fixation latencies toward sexual distractors, resulting in a low ACI. None of the covariates had a significant influence. We assume that both control groups already directed early attentional processes to the cognitive task. The ROC-analyses for both attentional control indices revealed good discrimination accuracy between pedophiles and non-pedophiles.

At first glance, this might be surprising, according to the classical definition of early attentional processes as to be mainly automatic and not susceptible to manipulation (48, 50). However, visual search studies have shown that top-down processes can be initiated very quickly in anticipation of irrelevant salient distractors (51). In a visual search task, Siebold et al. (52) found that initial saccades elicited after 250 ms were completely unaffected by salience and were increasingly led in line with task demands with increasing RT. In our study, the non-forensic control group’s shortest fixation latencies of about 400 ms were directed to the mental rotation figures (see Figure 2). According to Siebold et al. (52), already these early eye movements toward mental rotation figures could be driven by task demands. In contrast, pedophiles did not allocate the first fixation to mental rotation figures in the majority of cases. Instead, their first fixation latencies to a specific stimulus were nearly equally distributed between sexual distractors and mental rotation figures. This resulted in a high ACI, representing inferior attentional control. Even though it is possible to orient early eye movements according to task demands, pedophiles did not direct these to mental rotation figures.

Both forensic groups demonstrated poor cognitive performance, but differed significantly according to eye movements. Whereas eye movements of the forensic controls were similar to healthy controls, i.e., with good attentional control, this was not seen in pedophiles. Pedophiles demonstrated poor cognitive performance as well as inferior control of early eye movements. Hence, we suggest that there might be more behind this inferior control of early attentional processes in pedophiles than just cognitive performance parameters. These aspects will be discussed later, because they might be of general importance.

### Attentional Control under Cognitive Load – Late Attentional Processes

With respect to relative fixation time, all groups viewed mental rotation figures significantly longer than sexual distractors. Hence, all three groups followed the instruction, to look mostly at the mental rotation figures. However, the ACI was significantly lower for pedophiles compared to non-pedophiles. This was seen for both conditions, i.e., mental rotation tasks with an adult as well as with a child sexual distractor. None of the covariates had any influence. This could indicate lower attentional control in pedophiles than in non-pedophiles also regarding late attentional processes. ROC-analysis demonstrated moderate classification accuracy for both conditions.

Both forensic groups demonstrated poor cognitive performance, but they also differed according to the relative fixation time. Forensic controls exhibited a similar pattern to that of non-forensic controls with good attentional control. Interestingly, regarding this late attentional parameter, small but significant effects for sexual distractor category could be seen for control groups (see Figure 3; Table S2 in Supplementary Material). According to their sexual preference, adult distractors received more attention than child distractors. This is of special interest, because both control groups directed most of their late attentional processes to mental rotation figures. Thus, even both control groups demonstrated clear attentional control according to task demands a significant influence of sexually relevant distractors was found. Late attentional processes are susceptible to manipulations (48), but our results might indicate that the possibility to manipulate eye movements or behavior could decrease under cognitive load. In contrast, eye movement patterns of pedophiles did not fit with this assumption. Even though they viewed mental rotation figures significantly longer than sexual distractors, i.e., following task instructions, no sexual distractor effect was found (see Figure 3; Table S2 in Supplementary Material). Moreover, compared to control groups, longer fixation times to distractors and shorter fixation times to mental rotation figures resulted in a lower ACI, i.e., a low attentional control with respect to late attentional processes. In the following, we discuss some possible explanations for these results, firstly with respect to executive functions and secondly concerning further potentially influencing factors.

### Low Attentional Control to Sexual Distractors in Pedophiles and Executive Functioning

Impaired executive functions could be modulating factors concerning the low attentional control in our pedophilic subjects. According to the above described definitions of executive functions, we see the control of eye movements which were assessed in our study, as inhibitory functions at attentional level, i.e., interference control (20). Our participants had to inhibit their allocation of attention to salient sexual distractors, while concentrating on the cognitive task and initiating a response to the cognitive task. According to this, pedophiles showed a lower interference control in this task compared to both control groups. These results are in line with Eastvold et al. (23), who found an impaired interference control in pedophilic and non-pedophilic child molesters compared to non-sexual offenders. However, Eastvold et al. used the classical Stroop task, which contains neutral words and colors. We exclusively measured attentional control to sexual distractors. Hence, further studies should examine whether the results will change when other distractors will be used, for instance neutral or meaningless control distractors.

Furthermore, typical tests of executive functioning should be applied to additionally measure general executive functions, e.g., the traditional Stroop task to measure interference control toward neutral stimuli or the go/no-go task to measure inhibitory control at a simpler behavioral level. Since some studies reported impaired response inhibition in child molesters (24), the question is if both executive control functions, i.e., response inhibition and interference control are interrelated and if they correspond to attentional control functions in the sexual distractor task in the same sample. Factor analyses have found that inhibition of attention (interference control) and inhibition of action (response inhibition) are strongly correlated and fall along a single factor (53).

### Low Attentional Control to Sexual Distractors in Pedophiles – Other Influencing Factors

One critical question is whether there were other factors which could have modulated the task processing in our subjects. Models of selective attention and emotional interference propose that levels of attention are affected by the balance between bottom-up sensory stimulus-driven influences, such as salience, and top-down goal-directed influences, e.g., task demands (54). According to Oliveira et al. (55), additional factors could influence the association between the load of the main task and the distractor processing. First, the (subjective) relevance of the to-be ignored stimuli could determine its subsequent attention allocation. The second factor comprises the engagement in the main task, i.e., the subject’s motivation. Oliveira et al. (55) suggested that motivation applied to the main task functions to upregulate top-down control processes leading to more efficient task-requirements, thereby helping to diminish distractor processing. It is obvious that, in our task, sexual distractors should have an individually subjective relevance – all approaches to measure sexual interest are based on this assumption [see also Ref. (29)]. However, with respect to the high error rate for both forensic groups, we cannot exclude, that the task was too difficult for the forensic patients. The realization, that a task is too difficult for one’s self, could be frustrating and thus could decrease motivation to hold on task processing. But even though this could have happened for both forensic groups, they demonstrated different eye movements and a different attentional control. Thus, poor performance and potentially subsequent decreased motivation could not be the only reason for the low attentional control in pedophiles. Currently, we cannot disentangle if, independent of performance, pedophiles were not able or were not motivated to control their attention. Furthermore, based on the poor performance of the forensic control subjects, it is also conceivable, that they were motivated to avoid viewing sexual distractors rather than solving the task. One approach to answer those questions could be the above discussed integration of non-sexual control distractors in the current task.

### Impaired Attentional Control in a Sexual Distractor Task – Potential and Limitations

We suspect that the lower attentional control of pedophiles, regarding the sexual distractor task, could be related to impaired interference control. The latter belongs to executive functions.

However, several limitations have to be mentioned. First, we did not assess general executive functions in our participants in order to ask for associations between the tests. Further studies should include such kind of tests, for example response inhibition (go/no-go task), other measures of interference control (Stroop task), or cognitive flexibility (WCST). Furthermore, we exclusively measured attentional control to sexual distractors. To disentangle if the attentional control of the subjects is stimulus specific or not, additional non-sexual control distractors should be included. As discussed above, individual motivation to solve a task could modulate distractor processing. We did not explicitly assess motivation to solve the task. Furthermore, a task with individually adapted difficulty could probably help to resolve this point. Currently, we cannot disentangle if pedophiles were not able or were not motivated to control their eye movements. It is one advantage of our mental rotation task that the difficulty of the task can be systematically modulated by varying the angular disparity between the two figures (42). The characterization of sexual behavior, fantasies, sexual (deviant) urges and tendencies for sexual obsessiveness, and preoccupation could be helpful, to examine the association between those sexual characteristics and the eye movements in the sexual distractor task [e.g., Multiphasic Sex Inventory, MSI (56)]. Another critical point concerns the small number of participants, especially in the forensic control group. Further studies with larger groups should examine if the current results could be replicated.

Obviously, at the current level of development, we can only speculate about a potential clinical application of our experimental design. One of these speculations is based on the idea that inhibitory executive functions could be related to clinically important aspects of controllability, the capacity of self-control, and the severity of a paraphilic disorder.

Thus, if attentional control to sexual distractors, which we measured in the current design, could be linked to those clinically important characteristics, our design would potentially be helpful to assess self-control capacity and severity of paraphilia without directly asking the patients.

## Author Contributions

KJ has been involved in the development of the design, data collection, data analysis, and the preparation of the manuscript. PF has made important contributions to the development and programming of the design, data collection, data analysis, and the intensive revision of the manuscript. JH, HS, and RN have made important contributions to development of the design and data collection. JW gave substantial clinical support, supervised the clinical tests, and was involved in drafting and revision of the manuscript. JM supervised and supported the development of the idea and the design and was involved in the drafting and revision of the manuscript. All the authors have read and approved the final manuscript.

## Conflict of Interest Statement

The authors declare that the research was conducted in the absence of any commercial or financial relationships that could be construed as a potential conflict of interest.
