# Supplementary figures and images for: Impaired Attentional Control in Pedophiles in a Sexual Distractor Task
Source: Front Psychiatry. 2016 Dec 2;7:193. doi: 10.3389/fpsyt.2016.00193 (PMC5133255; doi:10.3389/fpsyt.2016.00193)

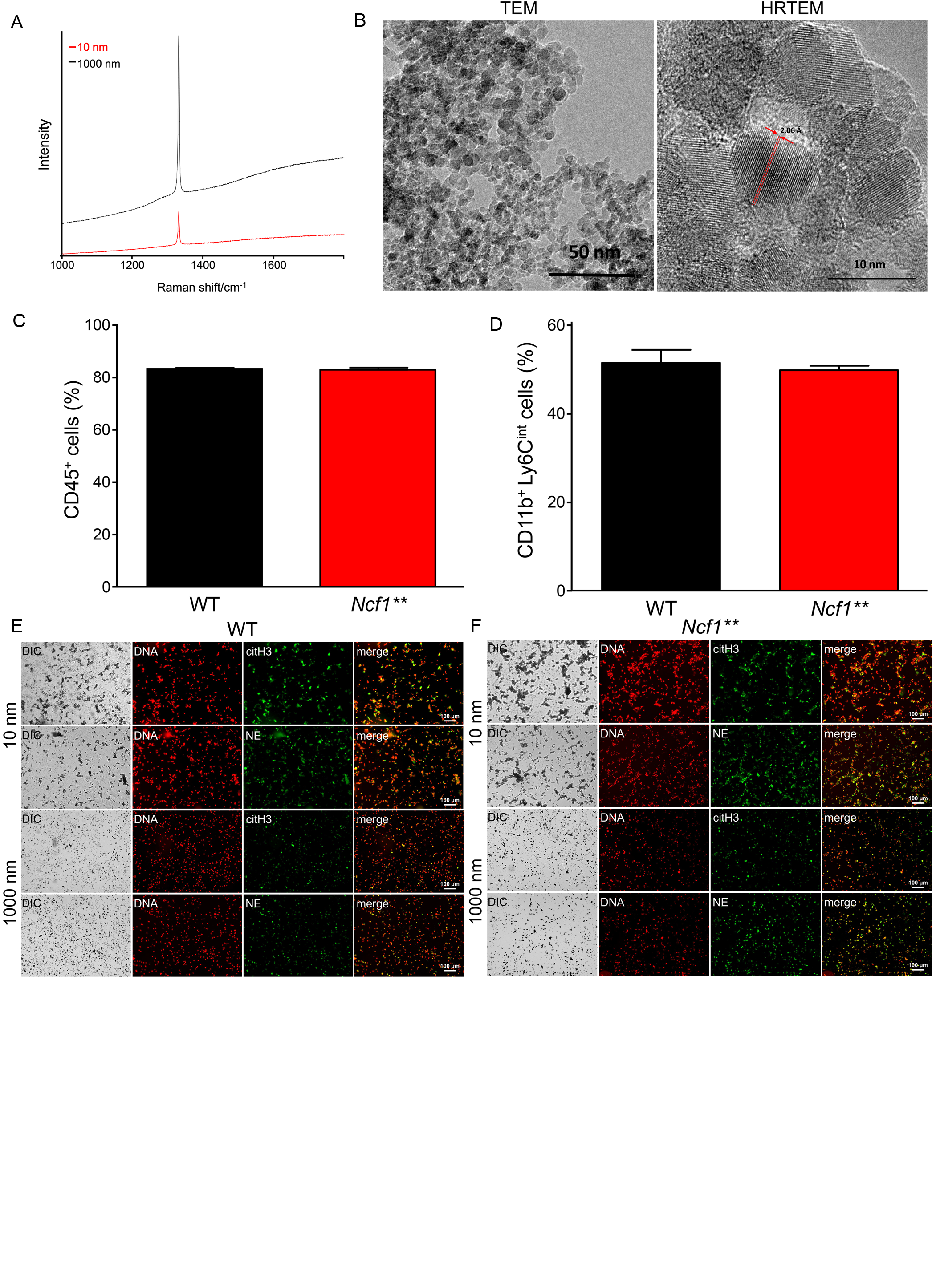

Supplement: Supplementary file 1 [file image_1.tif]
