# Supplementary material for: Impaired Attentional Control in Pedophiles in a Sexual Distractor Task
Source: Front Psychiatry. 2016 Dec 2;7:193. doi: 10.3389/fpsyt.2016.00193 (PMC5133255; doi:10.3389/fpsyt.2016.00193)
Supplement: Supplementary file 2 [file table_2.docx]

**Impaired attentional control in pedophiles in a sexual distractor task**

Kirsten Jordan, Peter Fromberger, Jakob von Herder, Henrike Steinkrauss, Rebekka Nemetschek, Joachim Witzel, Jürgen L. Müller

Author Note

Kirsten Jordan, Peter Fromberger, Jakob von Herder, Henrike Steinkrauss, Rebekka Nemetschek, Jürgen L. Müller, Department for Forensic Psychiatry and Psychotherapy,

Georg-August-University of Göttingen, Germany

Joachim Witzel, Central State Forensic Psychiatric Hospital of Saxony-Anhalt, Uchtspringe, Germany

Corresponding author:

Kirsten Jordan, kirsten.jordan@medizin.uni-goettingen.de

Georg-August University Goettingen, Department for Forensic Psychiatry and Psychotherapy,

Rosdorfer Weg 70, 37081 Goettingen

**Supplementary material**

**Table S2: Eye movements in the sexual distractor task: relative fixation time with respect to sexual distractor category and stimulus type.
 Results of the repeated measure GLM within groups are given.**

| Relative Fixation time [%] | | Pedophiles  N=22 | Forensic controls  N=7 | Non-forensic controls  N=50 |
| --- | --- | --- | --- | --- |
| Factor | **Effects** |  |  |  |
| Stimulus type | Main effect | ***F*(1, 21) = 65.51, *p* < .001, *ƞ^2^* = .76** | ***F*(1, 6) = 108.73, *p* < .001, *ƞ ^2^*= .95** | ***F*(1, 49) = 1468.9, *p* < .001, *ƞ ^2^*= .97** |
|  | Post hoc test | **Sexual distractors < mental rotation figures**  ***p* < .001** | **Sexual distractors < mental rotation figures**  ***p* < .001** | **Sexual distractors < mental rotation figures**  ***p* < .001** |
| Distractor category | Main effect | *F*(1, 21) = .41, *p* = .528, *ƞ^2^* = .02 | *F*(1, 6) = 3.89, *p* = .096, *ƞ^2^* = .39 | *F*(1, 49) = 3.53, *p* = .066, *ƞ^2^* = .07 |
|  | Post hoc test | n.a. | n.a. | n.a. |
| Distractor category * stimulus type | Interaction effect | *F*(1, 21) = .18, *p* = .672, *ƞ^2^* = .01 | ***F*(1, 6) = 6.06, *p* = .049, *ƞ^2^* = .50** | ***F*(1, 49) = 7.45, *p* = .009, *ƞ^2^* = .13** |
|  | Post hoc test | n.a. | Sexual distractors:  *F*(1, 6) = 1.43, *p* = .276, *ƞ^2^* = .19,  **Mental rotation (MR) figures:  *F*(1, 6) = 6.10, *p* = .049, *ƞ^2^* = .50**  **MR-figure with adult distractor < MR-figure with child distractor,**  ***p* = .049** | **Sexual distractors:**  ***F*(1, 49) = 11.42, *p* = .001, *ƞ^2^* = .19**  **Adult sexual distractor > child sexual distractors, *p* = .001**  Mental rotation figures:  *F*(1, 49) = 3.24, *p* = .078, *ƞ^2^* = .06 |

^1^Univariate repeated measure GLM with stimulus type (sexual distractor, mental rotation figure) and distractor category (adult, child) as within factors. Bonferroni corrected post hoc tests were applied
^2^n.a. not applicable
